# Digital Microfluidic Dynamic Culture of Mammalian Embryos on an Electrowetting on Dielectric (EWOD) Chip

**DOI:** 10.1371/journal.pone.0124196

**Published:** 2015-05-01

**Authors:** Hong-Yuan Huang, Hsien-Hua Shen, Chang-Hung Tien, Chin-Jung Li, Shih-Kang Fan, Cheng-Hsien Liu, Wen-Syang Hsu, Da-Jeng Yao

**Affiliations:** 1 Department of Obstetrics and Gynecology, Linkou Medical Center, Chang Gung Memorial Hospital, Taoyuan, Taiwan; 2 Department of Obstetrics and Gynecology, Chang Gung University and College of Medicine, Taoyuan, Taiwan; 3 Institute of Nanoengineering and Microsystem, National Tsing Hua University, Hsinchu, Taiwan; 4 Department of Mechanical Engineering, National Taiwan University, Taipei, Taiwan; 5 Department of Power Mechanical Engineering, National Tsing Hua University, Hsinchu, Taiwan; 6 Department of Mechanical Engineering, National Chiao Tung University, Hsinchu, Taiwan; Institute of Zoology, Chinese Academy of Sciences, CHINA

## Abstract

Current human fertilization *in vitro* (IVF) bypasses the female oviduct and manually inseminates, fertilizes and cultivates embryos in a static microdrop containing appropriate chemical compounds. A microfluidic microchannel system for IVF is considered to provide an improved *in-vivo*-mimicking environment to enhance the development in a culture system for an embryo before implantation. We demonstrate a novel digitalized microfluidic device powered with electrowetting on a dielectric (EWOD) to culture an embryo *in vitro* in a single droplet in a microfluidic environment to mimic the environment *in vivo* for development of the embryo and to culture the embryos with good development and live births. Our results show that the dynamic culture powered with EWOD can manipulate a single droplet containing one mouse embryo and culture to the blastocyst stage. The rate of embryo cleavage to a hatching blastocyst with a dynamic culture is significantly greater than that with a traditional static culture (*p*<0.05). The EWOD chip enhances the culture of mouse embryos in a dynamic environment. To test the reproductive outcome of the embryos collected from an EWOD chip as a culture system, we transferred embryos to pseudo-pregnant female mice and produced live births. These results demonstrate that an EWOD-based microfluidic device is capable of culturing mammalian embryos in a microfluidic biological manner, presaging future clinical application.

## Introduction

Fertilization *in vitro* (IVF) was an exciting scientific achievement of the twentieth century that maintains a great impact on human lives [1]. With the increasing clinical utilization of assisted reproductive technology (ART), scientists and clinicians have acquired insight into the basic biology of gametes and embryos and translated that knowledge into improved rates of success following assisted reproduction. These approaches aimed to improve embryo development *in vitro* have involved mostly the chemical composition of the culture media. Both conventional and sequential culture-medium systems have been refined; the development of high-quality blastocysts *in vitro* is common in clinical practice [2, 3].

Human IVF bypasses the female oviduct and manually inseminates, fertilizes and cultivates an embryo in an oil-covered static microdrop (μL) containing appropriate chemicals on a Petri dish and manipulated several times with manual pipetting. The labor-intensive manipulations of repeated washing and culture drops might introduce loss and prospective stress of temperature, pH, osmolality and light, and mechanical stress, on the embryos [4]. Although the culture platforms offer some advantages, the gametes and embryos rest on inert synthetic polymers and bathe in a significant volume of medium, in extreme contrast to what occurs *in vivo*. Furthermore, a cultured embryo experiences not only chemical (hormonal) but also prospective physical requirements that might be important factors in the continuing pursuit of improved conditions *in vitro* before implantation[5, 6].

Only recently have biomimetics and microfluidics become an approach to increase the rate of success of IVF. Additional implemental and analytical approaches and techniques are emerging; an examination of various novel culture platforms to explore the impact of physical and mechanical modifications on the embryo might assist in improving further the development *in vitro*[7–10]. Because of the comparable scales, microfluidics appear to offer a prospective means to improve other common procedures or approaches used within the laboratory for IVF, and elsewhere, near that which gametes and embryos experience *in vivo*. Microfluidics is applied to IVF in various aspects [11–13], including sperm processing, fertilization and embryo culture. Besides using the laminar flow characteristics of microfluidics to separate motile and non-motile sperm along a microchannel [14–18], microfluidic IVF is considered an improved means to mimic dynamic environments *in vivo* for cultured embryos[12].

All reported microfluidic IVF work has been done in continuous microchannels, which differ substantially from a microdrop currently used in clinical IVF [19, 20]. In our work, a single droplet is manipulated with electrodes deposited on the platform using electrowetting on a dielectric (EWOD) and dielectrophoresis (DEP) [21], simplifying the repeated washing and medium changes currently effected with manual pipetting. The EWOD system was derived from the electrowetting phenomenon that alters the contact angle of a liquid, so to enable a discrete droplet to be manipulated through reconfigurable paths or channels defined by electrode actuation. Compared with a continuous microfluidic channel, an EWOD digital microfluidic system has the advantages of minute sample and reagent volume, precise control of a droplet, small risk of contamination, small consumption of energy, low cost, rapid analysis, portability for diagnosis at a point of care, flexibility in integrating with a detector, multiple-step processes and simultaneous reaction [22–25]. In the present work, we describe a novel digital microfluidic device with EWOD to culture a mammalian embryo in a single droplet that provides a non-continuous microfluidic environment to mimic the environment *in vivo* for the development of an embryo and live births, presaging future clinical application.

## Materials and Methods

### Device construction

**Fig 1:**
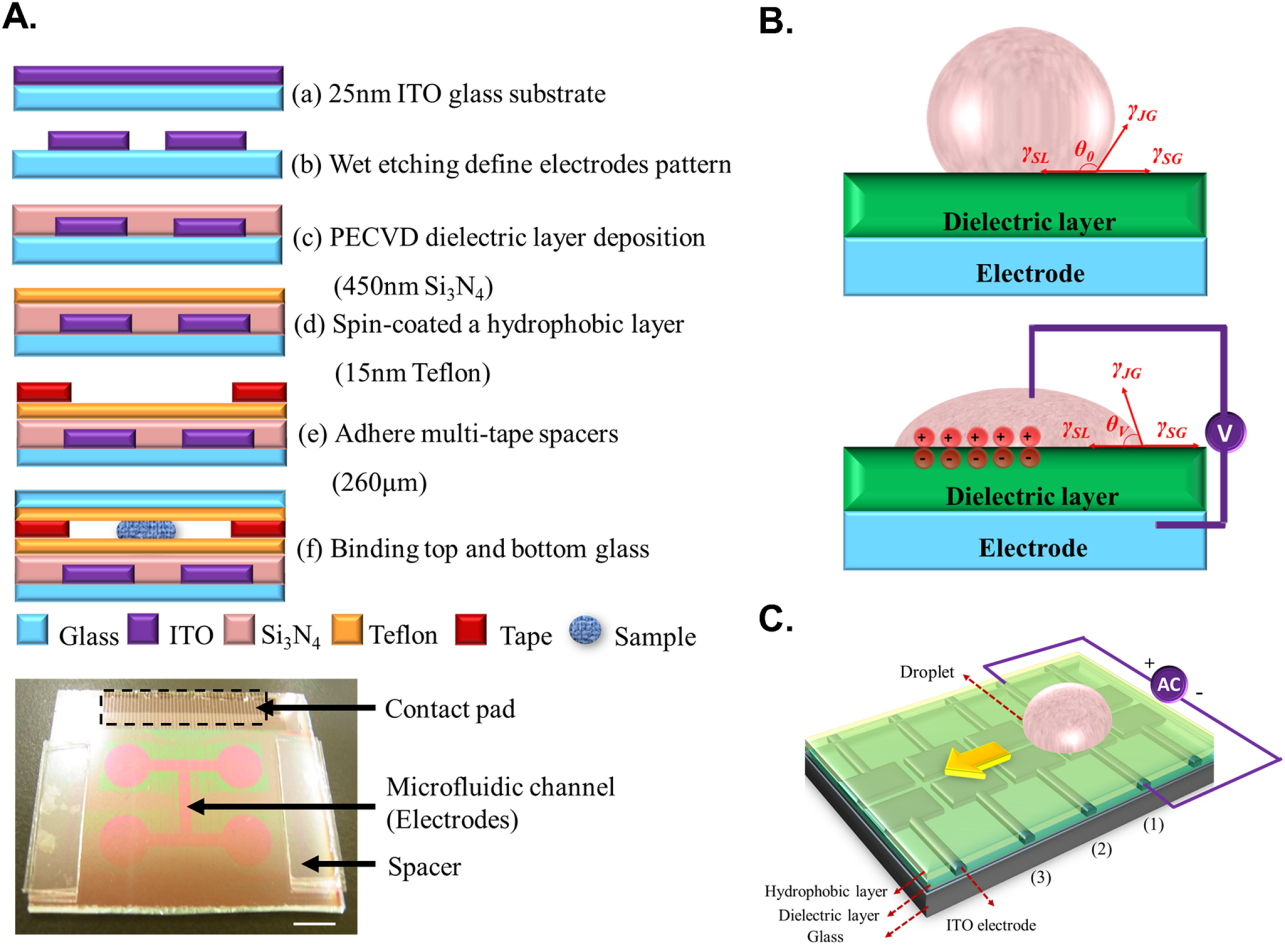
Scheme of EWOD chip design and fabrication and principle of EWOD. A. Description of EWOD chip fabrication and bottom plate of EWOD chips (white scale bar, 6 mm). B. EWOD is based on the variation of the wettability of liquids on a dielectric solid surface on altering the electric potential. The contact angle of the liquid is decreased from its initial contact angle when a voltage is applied. On releasing the electric potential, the contact angle of the liquid reverts to the initial contact angle. C. Scheme of droplet transport.

Devices were fabricated in the clean-room facilities of Institute of Nanoengineering and MicroSystems (iNEMS) in National Tsing Hua University (NTHU). The devices composed of coplanar-electrode type have a ground and five electrodes placed on the same plane, which bestows a flexibility to integrate further functionality with a top plate (Fig 1A). The gap between each electrode is 20 μm; the size of the control electrode is 0.75 mm × 1.5 mm. A digital microfluidic channel is made with an array of 15 × 2 control electrodes. The bottom plate has electrode arrays on indium tin oxide (ITO) glass patterned with photolithography and wet etching. Si_3_N_4_ (thickness 450 nm) and a spun-on thin film of glass (SOG) (thickness 200 nm) were deposited on ITO electrodes as a compound dielectric layer. Teflon was coated as a hydrophobic layer with contact angle 120°. The height of the microchannel space between the top and bottom plates was 260 μm with multi-tape as a spacer. The top plate was glass with a Teflon coating (thickness 20 nm) as a hydrophobic cap. In this research the microfluidic channel was pre-coated with pluronic F127 (0.08%) in a HTF medium for culturing an embryo to decrease biofouling and to enhance the protein stability [26].

### Device design with electrowetting-on-dielectric

EWOD is based on the variation of the wettability of liquids on a dielectric solid surface on altering the electric potential. The contact angle of the liquid is decreased from its initial contact angle when a voltage is applied. On releasing the electric potential, the contact angle of the liquid reverts to the initial contact angle (Fig 1B).

To create a droplet, we used four controllable electrodes and a reservoir electrode [27, 28]. We first activated the four controllable electrodes; the liquid was drawn by the reservoir electrode and moved towards electrode 1. When the controllable electrodes were full of the liquid, we deactivated electrodes 2 and 3, and activated electrode 1 and the reservoir electrode; the liquid produced a necking phenomenon, so creating a droplet on electrode 1 (Fig 1C). To manipulate the EWOD device, we applied an AC potential signal through a signal generator (Agilent 33220A, Agilent Technologies, Inc. Taiwan), which was amplified and output into the control circuit with a power amplifier (A.A. Lab Systems-AA303, Ramat-Gan, Israel). We used a computer to plan the route of the droplet transport and to output the signal to switch relays that turn on or off through a PCI digital I/O control card (NI USB-6509, National Instrument, Inc. Taiwan). The DMF chip was connected with the clamp (Yokowo, Micro Tech, Inc. Taiwan) via a cable to enable it to deliver an electric signal from the system to the clamp inside the cell-culture incubator. All this system must cooperate with an ordinary culture system with an incubator. Therefore we have created a hole on the wall of the incubator, and the cable was able to pass through the hole for connecting the electrical signal of the chip and system.

## Materials

The mouse experiments were approved by the Chang Gung Memorial Hospital (CGMH) Animal Care and Use Committee and conducted in accordance with the principles and procedures outlined in the CGMH Guidelines for the Care and Use of Laboratory Animals. Imprinting Control Region (ICR) mice were used in this research; imprinting is the mammalian phenomenon whereby the expression of a gene occurs only from one of two alleles, the other having been silenced. Female ICR mice were injected intra-peritoneally with PMSG (5 IU; Sigma, USA). 48 h after PMSG injection, the female mice were further injected intra-peritoneally with HCG (5 IU; Sigma, USA). Immediately after HCG injection, each female mouse was caged with a male mouse for mating (defined as day *E*_*0*_). 36 h post HCG injection, the female mice were sacrificed and the oviducts were dissected and placed in a Petri dish containing human tubal fluid medium (HTF) (Irvine Scientific, Santa Ana, CA, USA). Two-cell embryos for an embryonic development culture were released on tearing the oviducts (defined as day *E*_*1*.*5*_).

### Embryo loading and culture

In a microfluidic culture experiment, a single mouse embryo at the two-cell stage was cultured in a droplet (1 μL) of medium and covered with mineral culture oil (4 μL) (OVOIL, Vitrolife, Göteborg, Sweden). The medium droplet was driven with EWOD electrodes by automatic programming for three days. The automatic programming served to control the period and amplitude of an applied voltage. The medium droplets moved up to 100 times, each occasion lasting a few seconds. In every dynamic experiment, two medium droplets were placed in the EWOD chip. The development of the embryo in an EWOD system was individually observed on day 1 (day *E*_*2*.*5*_), day 2 (day *E*_*3*.*5*_) and day 3 (day *E*_*4*.*5*_) with a microscope. The control group was a culture in devices in static fluidics without a potential applied; embryo development was also recorded as a control.

### Embryo transfer

To test the reproductive outcome of the embryo collected from an EWOD chip embryo culture system, we transferred embryos to pseudo-pregnant female mice; these were ICR female recipient mice after animal balance at least six weeks of age that had been mated to sterile males the evening before the transfer. The females were tested for evidence of vaginal plugs after mating. Recovered blastocysts from each treatment group were transferred into the uterus of a separate pseudo-pregnant recipient using the method described previously [29, 30]. Briefly, the recipient mouse was anaesthetized (2.5% avertin per gram of body mass). The back of the recipient mouse was wiped with ethanol (70%) and then a small transverse incision (less than 1 cm) was made in the skin with line dissection scissors, about 1 cm to the left of the spinal cord, at the level of the last rib. The skin was slid to the left or right until the incision was over the ovary or fat pad, both of which were visible through the body wall. The body wall was picked up and a small incision made just over the ovary with fine dissection scissors to expose the oviduct, ovary and the upper part of the uterus. Embryos in a transfer medium were transferred into each uterine horn with a fine glass pipette. Each uterine horn was then returned to the body cavity; the skin incision was closed appropriately. At the end of the procedure, the mouse was returned to its cage and left undisturbed in a warm, quiet place. The mouse warm was kept warm with a heating pad placed over the top of its cage. The mouse recovered from the anaesthesia in approximately 20–30 minutes. All recipients were allowed to deliver and to raise pups, which were either raised to the weaning age to test their post-natal development or mated with their siblings from the same litter for future fertility testing.

### Data analysis

The difference (as percentage) of blastocyst development in each group was analyzed with statistical software (SPSS Inc., Chicago, IL). Data (as percentage) were analyzed with a χ^2^ test. A value *p* < 0.05 was considered to be statistically significant.

## Results

### Manipulation of a droplet in a medium

**Fig 2:**
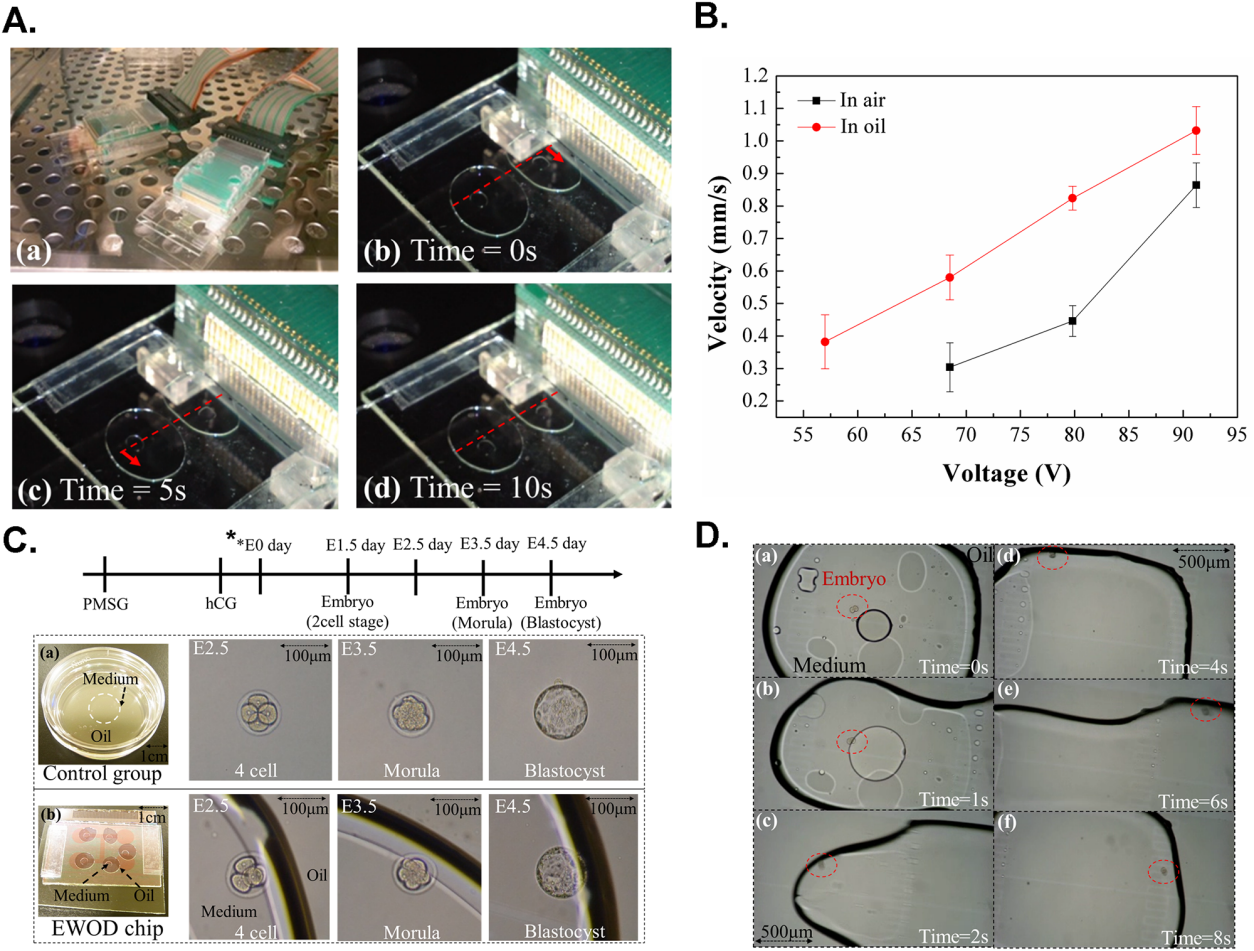
Operating the EWOD system for in vitro culture of embryo. A. Manipulating a droplet moving with an EWOD system. (a) The EWOD chip was placed in an incubator (b) Before motion, the droplet time = 0 s (c) The upper droplet moved 1.5 mm, right, time = 5 s (d) The lower droplet moved 1.5 mm, left, time = 10 s. B. Applied voltage versus velocity of droplets moving in air and in an oil-bath environment. C. Static embryo culture on a Petri dish and in an EWOD chip. The development of embryos in a EWOD chip was recorded as compatible relative to a traditional culture dish as control. D. Observation of an embryo in a moving droplet. Sequence images of embryo movement in a dynamic EWOD chip culture (a) before applied voltage. (b) The shape of droplet was altered with an applied voltage at the first electrode (c) The embryo was moving to the first electrode (d) The embryo stopped near the medium-oil interface (e) The embryo was moving rapidly when a applied voltage to the second electrode (f) The embryo was stopped near the medium-oil interface of the second electrode. *E day, the date after mating. Black arrow bar, 100 μm.

To mimic the environment flow *in vivo* and to take into account a shear stress effect, a decreased velocity of droplet motion was essential to assist an embryo to develop. When an EWOD electrode was turned on, a droplet moved from the original electrode to the next electrode. Our definition of the droplet velocity is derived from dividing the distance between two electrodes by the duration of a droplet to reach the nearby electrode. In a dynamic cell culture, the droplet velocity was controlled in range 0.3 ~ 1 mm s^-1^ to mimic the movement of an cleavage embryo in an oviduct (Fig 2A). To maintain the droplet moving fluently, we applied an electric potential (60~68.5 V _RMS_) at 500 Hz. The Eq (1) describing the velocity dependence of the contact angle [31]:

$$v=2k_{0}\lambda \text{sinh}(\frac{YLV}{2nk_{B}T}[\text{cos}\theta_{0}+\frac{\varepsilon_{0}\varepsilon_{r}}{2dYLV}V^{2}-\text{cos}\theta_{E}])$$ (1)

Where cosθ_E_ is the dynamic contact angle in the presence of the electric field. The γLV is the liquid-vapor surface energies respectively, d is the thickness of the solid insulator, ε_r_ and ε_0_ are the dielectric constant of the dielectric and the air, k_B_ is Boltzmann’s constant, *T* is the temperature, and there are *n* adsorption sites per unit area. The overall speed v is determined by the frequency k_0_ and length λ of these displacements. Reasonably good curve-fits to the experimental data were obtained with this equation for values of λ and k_0_ which were independent of the electric field. The contact angle hysteresis can be modeled as a negative energy constant in the sinh argument expressed as an effective voltage threshold V_T_. The Eq (2) can be obtained as:

$$v=2k_{0}\lambda \text{sinh}(\frac{\varepsilon_{0}\varepsilon_{r}}{4dnk_{B}T}[V^{2}-V_{T}^{2}])$$ (2)

In this research, the relation between an applied voltage and the droplet velocity of the digital microfluidic (DMF) system has been measured and shown in Fig 2B. In each experiments, we try to make the operational voltage as low as possible to reduce the effect of the electric field to the development of an embryo. However, the lowest droplet operation voltage depends on different DMF chips. Sometimes when the voltage was lower than 60V, the droplets manipulation would be unstable.

To demonstrate that the embryo development cultured on an EWOD chip was compatible with culture on a traditional culture dish, we cultured a single mouse embryo at the two-cell stage in a medium droplet (1 μL), covered with culture oil (4 μL) on an EWOD chip in static fluidics without an applied potential and in a traditional culture dish. The development of the embryo was observed individually on day 1 (*E*_*2*.*5*_), day 2 (*E*_*3*.*5*_) and day 3 (*E*_*4*.*5*_) with a microscope. The development of embryos in an EWOD chip was recorded as compatible with that of the control in a traditional culture dish (Fig 2C).

### Embryo development in a dynamic culture

The embryo was moved with droplet manipulation powered by EWOD. Fig 2D shows the result of the embryo in a dynamic environment using an EWOD chip. The velocity and shear stress of the embryo were affected by the position in the droplet. The embryo was initially in the middle, moving slowly with the fluid when the droplet was driven. The embryo ended at the medium-oil interface after the droplet crossed to the next electrode. The position near the interface caused the embryo to move rapidly: the embryo moved approximately 1.5 mm in 2 s when the droplet was driven. The dynamic fluid environment was demonstrated with the EWOD chip of which the result showed the embryo to move on manipulating a droplet. A video clip appears in S1 Video.

**Fig 3:**
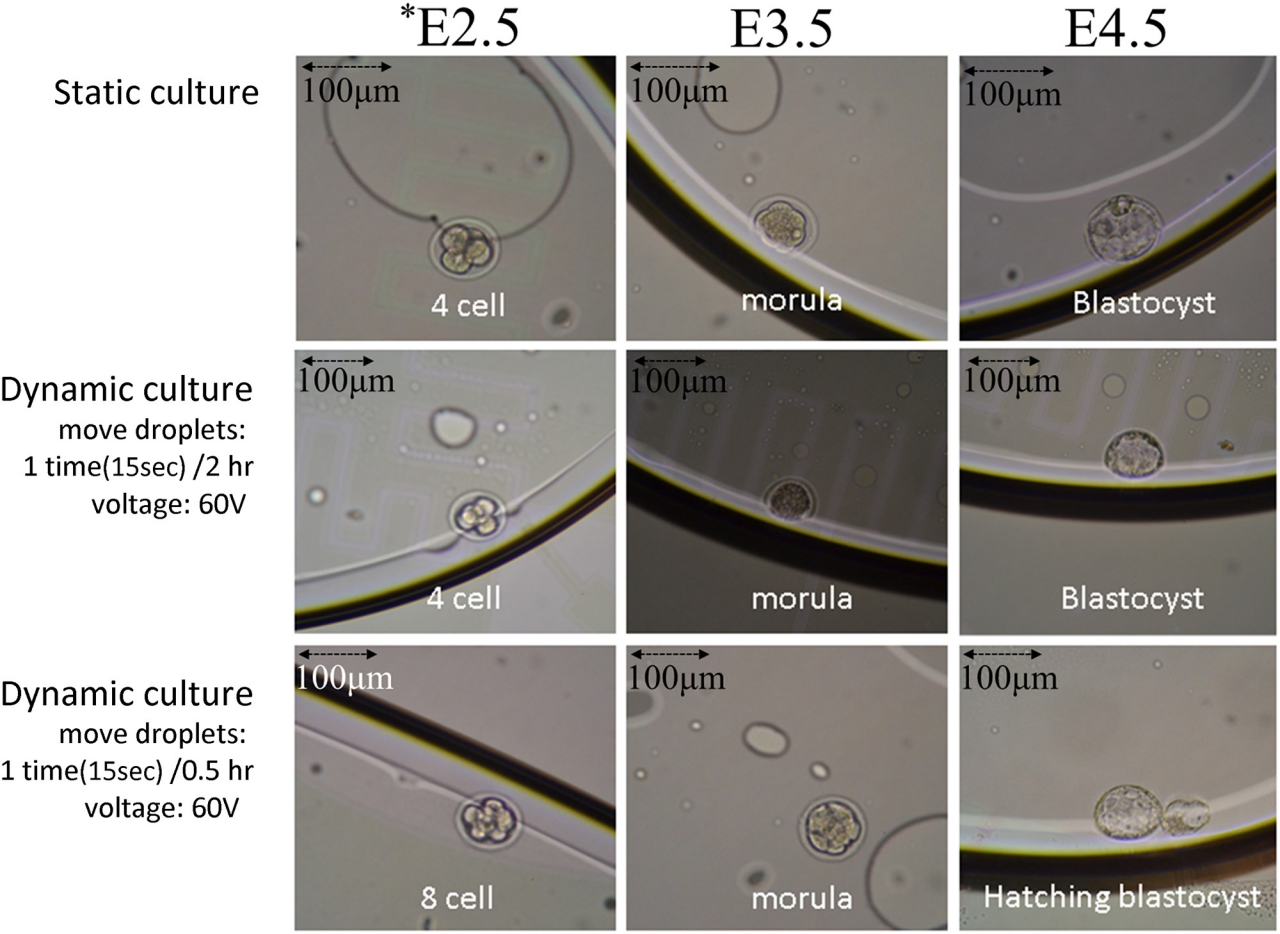
Dynamic embryo culture in EWOD chips. Embryo culture development on the EWOD system, The upper panel is static culture on the EWOD chip: from 4-cell stage to expanded blastocyst stage; The middle and lower panels are dynamic culture with different velocity of droplets: from 4 or 8-cell stage, early blastocyst stage to hatching blastocyst stage. The embryo development in dynamic culture with the droplet velocity (15 sec /0.5 hr) was hatching earlier in comparison to the embryo in static culture. *E day, the date after mating. Black arrow bar, 100 μm.

The embryo development of dynamic culture is shown in Fig 3. The speed of cleavage of an embryo in a dynamic culture was greater than that in a static culture. The development of an embryo in droplet with different velocity in a EWOD system was hatching earlier than those embryos in the static control-group culture.

### Embryo development and blastocyst quality

**Fig 4:**
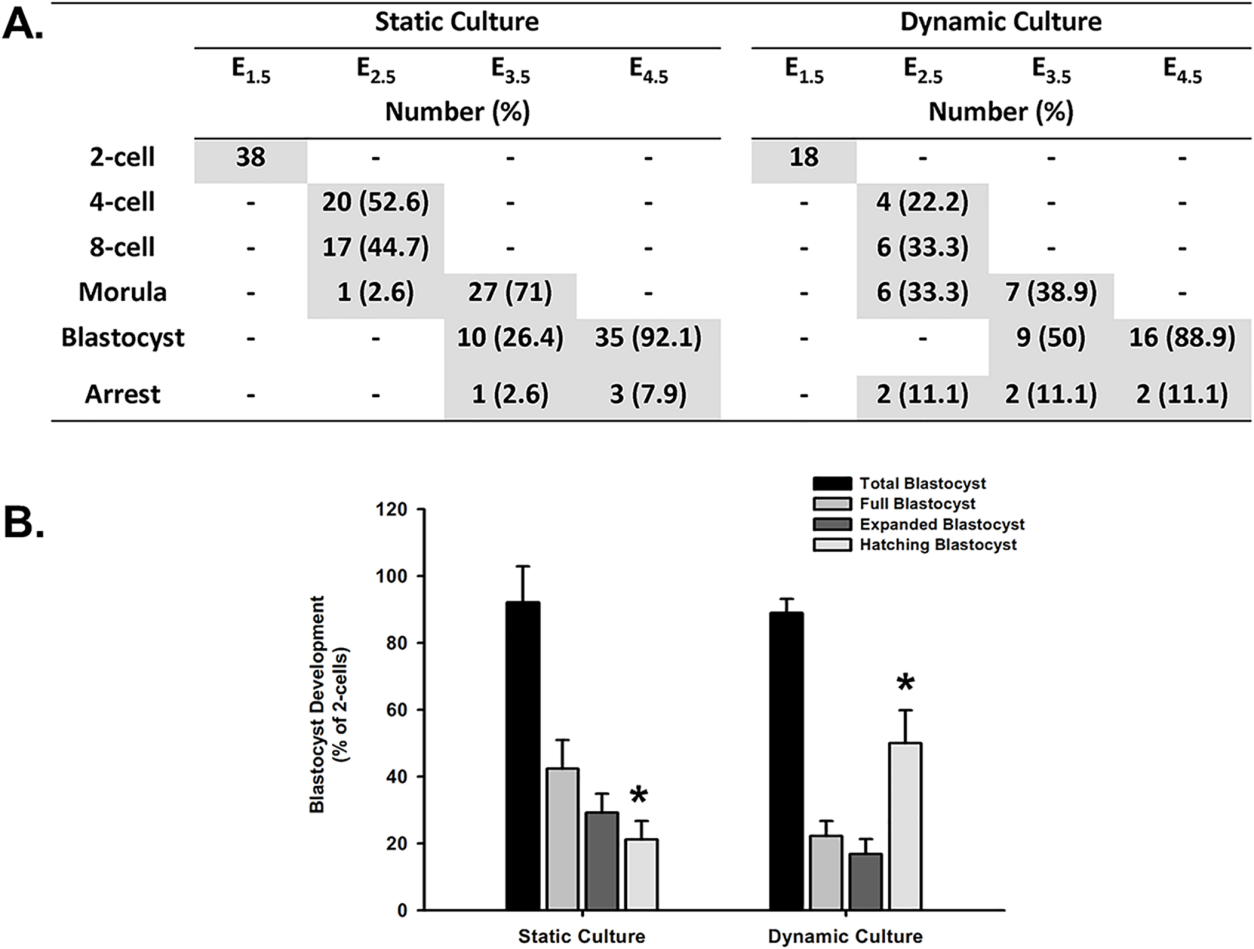
The embryo development and blastocyst quality of dynamic culture on EWOD chip. A. The statistics result of blastocyst development in EWOD dynamic culture (n = 18) in comparison to static culture (n = 38). Most embryos were successfully cleaved to a blastocyst stage. B. The blastocysts collected on day 3 (*E*_*4*.*5*_) is further classified according the morphology grading. A significantly higher rate of hatching blastocyst was demonstrated in dynamic culture in comparison to static culture (p = 0.028). *, Chi-square test, p<0.05. Values are mean ±standard error of measurements.

The statistical result of the embryo development in an EWOD dynamic culture (n = 18) on comparison with a static culture (n = 38) is shown in Fig 4. Most embryos were cleaved to a blastocyst stage (88.9±4.2% vs. 92.1±10.8%, respectively) as shown in Fig 4A. The blastocysts is further classified day 3 (*E*_*4*.*5*_) as full blastocyst (22.2±4.5% vs. 42.3±8.6%), expanded blastocyst (16.7±4.6% vs. 29.1±5.7%) and hatching blastocyst (50±9.8% vs. 21.2±5.5%, *p* = 0.028) as shown in Fig 4B.

### Blastocyst recovery from EWOD chip and viability after embryo transfer

**Fig 5:**
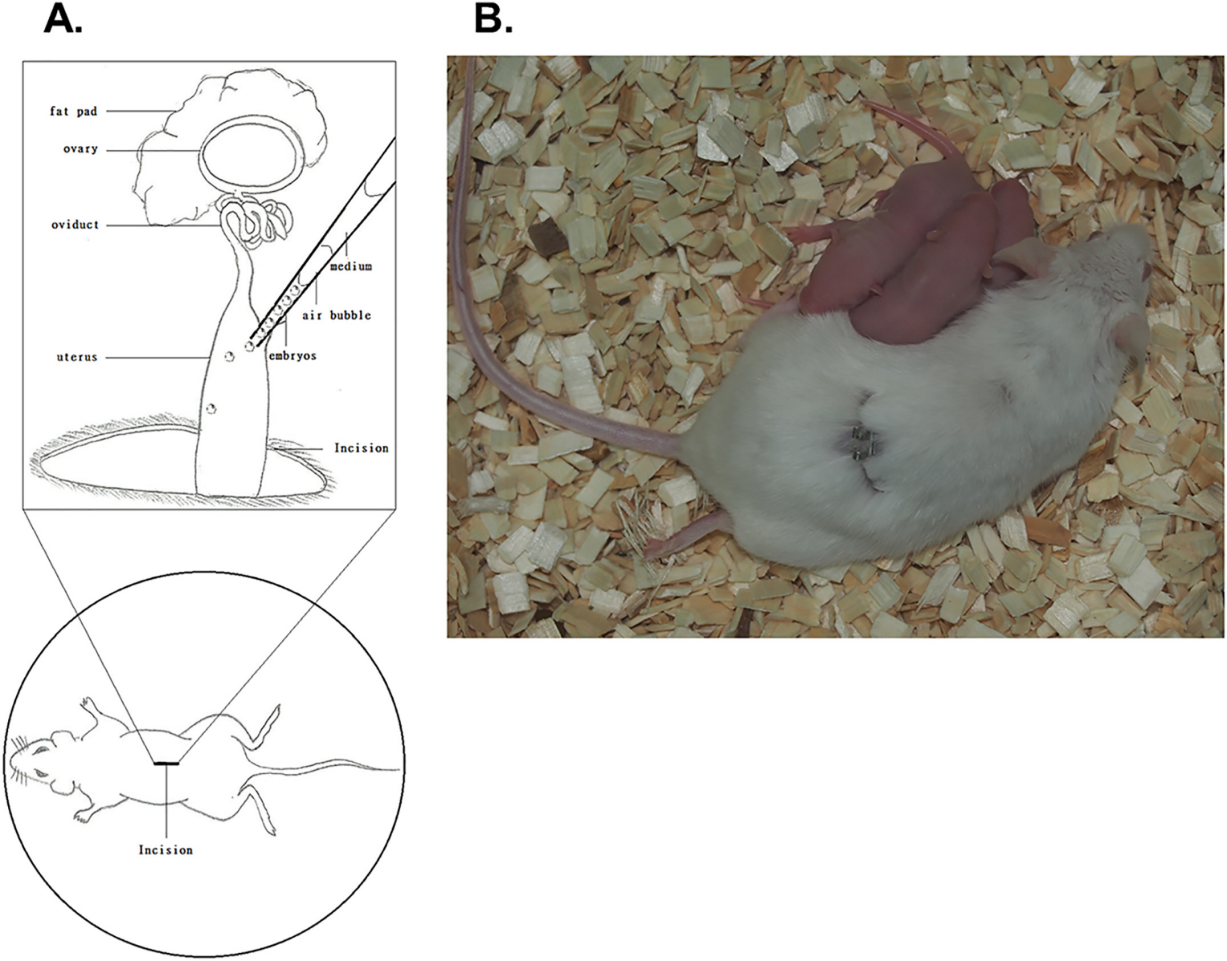
Embryo transfer from EWOD embryos to pseudo-pregnant female mice. Panel A, Preparation of recipient mice for embryo transfer. Panel B, Pups was born after transfer of embryo cultured from EWOD. The recipients were allowed to deliver and raise pups.

To test the reproductive outcome of the embryos successfully collected from an EWOD chip, we transferred the EWOD embryos to pseudo-pregnant female mice and produced live births. All blastocysts that developed from the EWOD and control groups after culture in vitro simultaneously were transferred into surrogate recipients (up to six embryos transferred individually for three experiments). The recipients became pregnant and were allowed to deliver and raise pups that were either raised to the weaning age to verify their post-natal development or mated with their siblings from the same litter for fertility testing (Fig 5). Of the blastocyst from EWOD embryos, 19 blastocysts were transferred to 3 pseudopregnant mice, of which 3 had pups. A total of 10 pups were born, representing 50.6% of the transferred blastocysts from EWOD group. All of the 10 pups born from EWOD group were grown up to 3 weeks with normal appearance.

## Discussion

According to our results, the culture *in vitro* of a mouse embryo using a EWOD system to provide a dynamic fluid environment is capable of culturing mammalian embryos in a microfluidic biological manner. A comparison of the results of the dynamic culture with the static culture also proves that the dynamic culture in the EWOD system enhanced the cleavage and development of a mouse embryo to the preimplantation stage.

For the preimplantation embryo cleavage *in vivo* in the oviduct and ultimately developed in the uterus, contractions of the inner muscle of the oviduct and movement of epithelial cell cilia cause an embryo to move continually. To mimic the dynamic fluid environment *in vivo*, the EWOD system required a decreased velocity of the droplet. The embryo velocity in a droplet is random; the EWOD system provided a dynamic environment, but a fixed velocity of embryo movement was unavailable. The gradients of potassium, calcium and oxygen in a medium droplet were disrupted, however, through the manipulation of droplets that established a suitable environment for embryo development.

Microfluidics is a promising new technique. Increased attention is thus being devoted to microfluidics in the field of reproductive medicine. Microfluidics, based on the physical principles of fluid behavior in a microenvironment, serve widely for applications in chemistry and molecular biology [32–35]. Many advances in nanotechnology and MEMS are applied to study biological systems. Microfluidic devices are being developed for biological assays with the potential to perform analysis with efficient throughput, to utilize samples or reagents in small amounts and for analysis of single cells [36, 37].

Microfluidic systems have been variously designed for IVF [38–40]. Sano *et al*. developed a microfluidic sperm sorter (MFSS) for porcine IVF[41], designed to isolate motile human sperm with laminar flows. Brief co-culture of porcine oocytes with sperm gradually accumulated in the MFSS chamber improved the efficiency of producing monospermic fertilized embryos and blastocysts; efficiencies were significantly affected by the oocyte location within the chamber. In a novel microwell-structured microfluidic device integrating trapping of a single oocyte, IVF and embryo culture reported by Han et al.[12], a microwell array served to capture and to hold individual oocytes during the flow of oocyte and sperm loading, medium substitution and debris cleaning. Fertilization was achieved in the microfluidic devices with rates of fertilization similar to those of standard oil-covered drops in Petri dishes.

Embryos were cultured to blastocyst stages in devices with developmental status individually monitored and tracked, but the reported results of microfluidic IVF are still preliminary and limited. Some results are even contradictory. For example, Hickman *et al*. reported that the continuous media perfusion to embryos within a microchannel resulted in poor embryo development for flow rates across a range [42]. In general, researchers speculated that the refreshing of nutrition and removal of waste increased the rate of development. A small rate of development was attributed to the shear stress and continual removal of growth-promoting autocrine factors [43]. To achieve a more reproducible and biomimetic condition, further investigation must include the influences of the microchannel dimensions and the flow rates. A computerized microfluidic device for embryo culture and assay in real time is required that can perform automated periodic analyses of embryo metabolism [44].

The microfluidic IVF system also possesses several major advantages over a conventional culture system. With microfluidics, fewer sperm are needed because of the smaller medium volume. The optimal concentration of sperm was in range 2~8x10^4^ sperm/mL, significantly less than the standard IVF (1×10^6^ sperm/mL) in tested microfluidic channels [15].

Raty *et al*. found that embryos cultured in microchannels filled with static media exhibited a greater rate of cleavage and produced more blastocysts than control microdrops [45]. Some authors indicated that the polyspermic penetration was decreased in microchannels [46]. The development of mouse embryos is advanced within microfluidic devices with media flow relative to static devices, resulting in increased rates of blastocyst formation and hatching [47]. With microfabrication, versatile microfluidic devices are practicable for integrated and automated IVF functions [12], but the reported results of microfluidic IVF are still preliminary and limited; some are even contradictory.

EWOD (electrowetting on a dielectric) is a microfluidic strategy that exploits surface tension as a means to manipulate liquid droplets at the current stage [48, 49]. An electric voltage is applied; the electric charge alters the Gibbs energy on the dielectric surface, causing an altered wettability of the surface and contact angle of the droplet [50]. The development of an EWOD digitalized microfluidic system capable of transporting oocytes or embryos to a desired location, producing mammalian embryos *in vitro* and chemical or mechanical manipulations, is highly desirable.

We applied our expertise of digital microfluidics to demonstrate dynamic IVF, preserving the merit of the microdrop method but enhancing its capability with controllable and dynamic droplets. Our study of dynamic IVF on a digital microfluidic platform based on the preceding microdrop IVF studies, especially the developed methods, is the first. Several features have been demonstrated, especially the culture of the embryo to the ultimate stage (blastocyst), developed earlier than for the traditional culture group. The droplets containing the embryo are manipulated with electrodes deposited on the platform using EWOD and dielectrophoresis [21], simplifying the repeated washing and medium changes currently involved with manual pipetting. In contrast with the static microdrop method, the movable droplets generate internal flows that decrease the point depletion or accumulation of substances in the medium with possible detrimental effects on the embryo development [19]. The gentle agitation of a medium and embryos would enhance outcomes by improved mimicking of the fluid-mechanical stimulation that embryos experience *in vivo* from ciliary beating and oviductal contraction [44]. Furthermore, the embryos can be moved with the droplet at an appropriate speed as in the oviduct [20]. The droplets serve not only as an isolated environment for embryos but also to perform sophisticated fluidic functions for diagnostic protocols (embryo selection or metabolic profile).

In research on EWOD droplet manipulation, workers have tried to make the applied voltage minimal. Water droplets can be manipulated with a small voltage [51]; some researchers used varied dielectric layer materials to decrease the voltage for droplet manipulation [52]. Lin et al. used 17 V_rms_ for manipulation of a water droplet [53], but a greater voltage is necessary for manipulation of a cell culture medium because it contains abundant electrolyte and biomolecules. Cell-based EWOD experiments typically require 100–300 V_rms_ to manipulate droplets [54–56]. In EWOD devices, the voltage is applied to induce an electric double layer in a dielectric layer and to alter the contact angle between a hydrophobic layer and a droplet. Even though electric charge cannot pass through the dielectric layer into a droplet on the EWOD chip, we decreased the voltage to 68 V_rms_ for a stable culture of an EWOD dynamic embryo and tried to discover the influence of EWOD manipulation of electrical droplets on the embryo development.

Some concerns are addressed on the effect of a EWOD system on embryogenesis. Embryogenesis is a complicated process according to which a fertilized egg develops into a multicellular organism, during which fundamental biological self organization occurs. The application of electric fields at small frequency to cells has a long and contentious history. All work indicates that developing embryos produce endogenous currents involved during embryonic development. Researchers also discussed a possible mechanism of a small electric field on isolated cells [57–60]. The electric field has a potential as a research tool for various biotechnological experiments with mammalian gametes and embryos. A parthenogenetic development of oocytes induced with an electric field has been performed in mammalian species [61, 62]. Electric activation can facilitate the fertilization and early embryonic development after ICSI (Intracytoplasmic Sperm Injection) in human beings [63, 64]. Popova *et al*. used mouse and rat embryos as models to investigate the sensitivity of oocytes and embryos to electric fields [65]; these authors suggested that electrical activation might facilitate fertilization and early embryonic development after ICSI in human beings. As electric stimulation is known to enhance protein and DNA synthesis, to stimulate neuronal cells and to differentiate the migration of neural crest cells, this work was planned to observe the developmental differences of Xenopus embryos exposed to electric fields during embryogenesis. All this work might provide some explanation of the dynamic cultural result relative to a static cultural result that proved a dynamic culture in the EWOD system to enhance the cleavage and development of a mouse embryo and to accelerate the growth to the blastocyst stage. As little is known about the sensitivity to electric fields of mammalian embryos of various species and at various stages of the early pre-implantation development, further investigation is necessary, including the influences of the microchannel dimensions and the flow rates, to achieve a more reproducible and biomimetic condition.

## Conclusion

We describe a digital microfluidic device that shows a feasibility to perform a dynamic embryo culture on an EWOD platform. We manipulated the function of a medium droplet containing a bio-sample in an EWOD chip with a modified complicated dielectric layer and a voltage in a culture incubator. The devolvement and the reproductive potential of the dynamic cultured embryo in an EWOD chip is compared with a traditional culture system. The device allows a capability of culturing mammalian embryos in a microfluidic biological manner. Further development might facilitate a new protocol embryo development and clinical IVF practice.

## Supporting Information

S1 Video Shows digital microfluidic dynamic culture of mouse embryo using EWOD chip. (WMV)
